# Targeting the X Chromosome during Spermatogenesis Induces Y Chromosome Transmission Ratio Distortion and Early Dominant Embryo Lethality in *Anopheles gambiae*

**DOI:** 10.1371/journal.pgen.1000291

**Published:** 2008-12-05

**Authors:** Nikolai Windbichler, Philippos Aris Papathanos, Andrea Crisanti

**Affiliations:** Faculty of Life Science, Imperial College London, London, United Kingdom; Princeton University, Howard Hughes Medical Institute, United States of America

## Abstract

We have exploited the high selectivity of the homing endonuclease I-PpoI for the X-linked *Anopheles gambiae* 28S ribosomal genes to selectively target X chromosome carrying spermatozoa. Our data demonstrated that in heterozygous males, the expression of I-PpoI in the testes induced a strong bias toward Y chromosome–carrying spermatozoa. Notably, these male mosquitoes also induced complete early dominant embryo lethality in crosses with wild-type females. Morphological and molecular data indicated that all spermatozoa, irrespectively of the inheritance of the transgene, carried a substantial amount of I-PpoI protein that could attack the maternally inherited chromosome X of the embryo. Besides the obvious implications for implementing vector control measures, our data demonstrated the feasibility of generating synthetic sex distorters and revealed the intriguing possibility of manipulating maternally inherited genes using wild-type sperm cells carrying engineered endonucleases.

## Author Summary

*A. gambiae* mosquitoes are the main vectors of human malaria. The inadequacy of existing control measures for these mosquitoes has prompted research into methods for genetic control. We have genetically engineered *A. gambiae* mosquitoes to express, during spermatozoa development, an enzyme that selectively cuts a DNA sequence present only on a family of essential genes located on the X chromosome. We found that in heterozygous male mosquitoes, this genetic modification induced complete early dominant embryo lethality in crosses with wild-type females. All spermatozoa from these males, including those not containing the genetic modification, carried the chromosome X cutting enzyme that could attack the maternally inherited X chromosome of the embryo. Furthermore, this genetic modification introduced a strong, negative bias toward X chromosome–carrying spermatozoa. These transgenic mosquitoes fulfil a number of requirements for implementing vector control measures based on genetic sterility, but our data also demonstrate the feasibility of generating synthetic sex distorters and reveal the possibility of manipulating maternally inherited genes using wild-type sperm cells carrying enzymes designed to attack selected maternal DNA sequences.

## Introduction

Mosquitoes represent a major and global cause of human suffering due to the infectious agents they transmit. About two million people die from mosquito-borne diseases every year. These include parasitic infection, i.e. malaria and filariasis and viral diseases such as dengue, encephalitis and yellow fever. Malaria alone, transmitted exclusively by *Anopheles* mosquitoes carrying *Plasmodium* protozoan parasites, causes the death of more than a million people each year, most of which are occurring in sub-Saharan Africa [1]. Rather than being under control, the threat represented by mosquitoes is increasing due to the inadequacy of existing control measures in the developing world and the progressive spread of insecticide-resistant insects [2].

Gene manipulation technologies promise to dramatically enhance the development of novel control measures against vector-borne diseases [3]. Different approaches are being investigated including the development of disease-refractory mosquitoes to implement population replacement strategies [4]–[8]. Genetic sterility, genetic drive systems or the release of insects carrying a dominant lethal gene (RIDL) have been suggested as possible strategies to reduce population density [9]–[14]. A number of reports have shown how genetically manipulated mosquitoes can provide valuable solutions to overcome ineffective, costly and time-consuming steps that have previously hampered vector control measures involving the sterile insect technique [15] (SIT). These include the use of genetic markers to monitor both male dispersal and mating competitiveness and the separation of male and female mosquito larvae at an early developmental stage to address the requirement to release only male mosquitoes, as females contribute to disease transmission [16]. An inducible genetic sterility system, designed to overcome the fitness cost associated with chemical and irradiation sterilization, has been developed in *Drosophila* as a proof of principle [17].

A novel mechanism has recently been proposed to distort the sex ratio in natural populations based on the use of engineered mosquitoes expressing a homing endonuclease enzyme targeting X chromosome carrying spermatozoa, thereby generating an excess of spermatozoa carrying chromosome Y [13],[18]. It has long been recognized that if the Y chromosome were to show transmission ratio distortion and spread in a population, then the sex ratio would become male biased and the population could ultimately go extinct [19],[20]. Natural driving Y chromosomes in *Aedes aegypti* and *Culex pipiens* have been described and can produce extreme sex ratios of more than 90% males in each generation [21]. Although the molecular details of how these distorters act are unknown, cytological evidence suggests that they are associated with breaks in the X chromosome during male meiosis I [22],[23]. A similar system for sex ratio distortion could be artificially created using the I-PpoI homing endonuclease: this enzyme has been shown to selectively cleave the ribosomal rDNA repeats in the *A. gambiae* Sua 4.0 cell line, leading to nucleolar fragmentation and cell death [18]. In several anopheline species, including at least two members of the *A. gambiae* complex, the rDNA repeats are exclusively located in the centromeric region of chromosome X [24]–[26]. Accordingly, the expression of I-PpoI during spermatogenesis is anticipated to incapacitate X chromosome carrying spermatozoa and induce sex ratio distortion. This mechanism would provide a formidable tool to distort the ratio in favour of males, thereby leading to the reduction or eradication of field populations.

We have engineered male *A. gambiae* mosquitoes to express, during spermatogenesis, the I-PpoI homing endonuclease as a fusion protein with the eGFP fluorescent marker with the aim of inducing sex ratio distortion, combined with the expression of an early developmental marker for sexing. We report here on the unique phenotype of these transgenic mosquitoes in terms of fertility and transmission ratio distortion of the sex chromosomes.

## Results

### Identification of I-PpoI Recognition Sequences in the *A. gambiae* Genome

The I-PpoI recognition sequence consists of a 15bp core motif flanked by a number of additional nucleotides that also contribute to the overall efficiency of the endonuclease binding and cleavage activity [27]. We searched the *A. gambiae* genome for the presence of the cognate I-PpoI recognition site. This analysis revealed no match to the wild type 15 base pair core recognition sequence outside the rDNA genes which were recently mapped to contig AAAB01008976 of the chromosome X of *A. gambiae* [28]. We also screened the genome for sequences matching a number of previously described recognition sequence variants that were identified by in vitro selection to be efficiently cut by I-PpoI [29]. None of these variants were found in the *A. gambiae* genome, even when taking into account only the 15bp core recognition sequence. Recently a complete specificity profile of I-PpoI has been established (N. Nomura, personal communication). Utilizing this profile we screened the *A. gambiae* genome for any recognition site variants predicted to be cut by I-PpoI with high efficiency. Again this analysis did not reveal matches in the *A. gambiae* genome assembly. We therefore concluded that the X-linked multi-copy rDNA locus, containing the complete I-PpoI core and flanking recognition sequence, would be the main predicted target locus of I-PpoI in the *A. gambiae* genome.

### Generation of Transgenic Mosquitoes Expressing I-PpoI during Spermatogenesis

**Figure 1:**
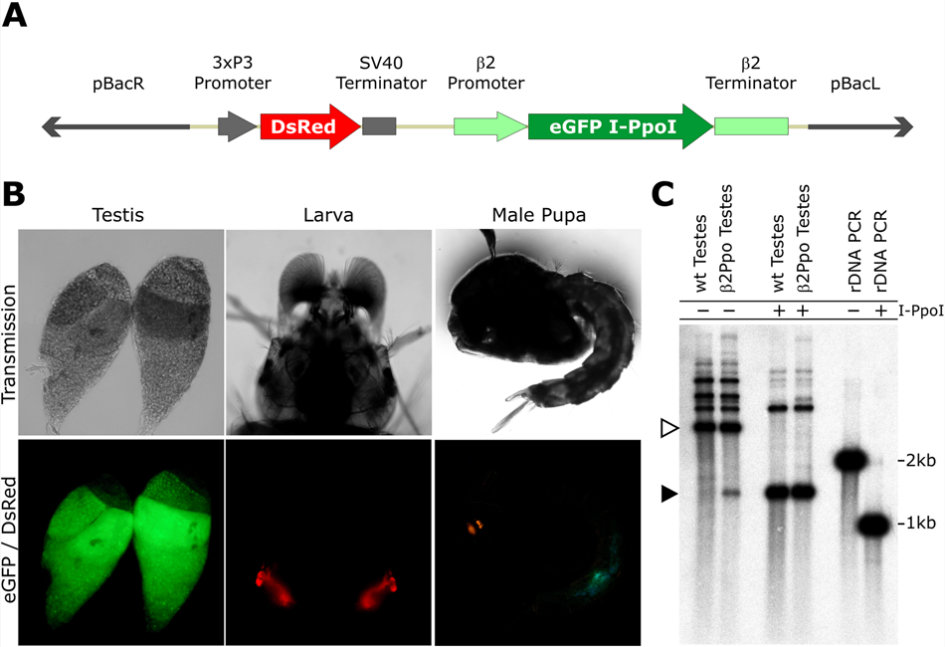
Transformation construct and expression of I-PpoI in the testes of transgenic mosquitoes. (A) Schematic representation of the construct pBac{3xP3-DsRed}β2-eGFP::I-PpoI containing the left and right piggyBac inverted repeats (pBacR,L); the Pax promoter (3xP3) driving the DsRed marker gene; and the eGFP::I-PpoI effector gene (eGFP I-PpoI) under the control of β2 tubulin promoter and terminator (β2). (B) Transmission and fluorescent images of dissected adult testes, larval head and pupa of β2Ppo1 male mosquitoes. (C) Southern blot analysis of the 28S ribosomal DNA locus. DNA from testes of WT (lanes 1 and 3) and β2Ppo1 males (lanes 2 and 4) was digested with the endonuclease ClaI *in vitro* and hybridized with a probe encompassing the 28S ribosomal gene (Figure S1). As control both the DNA extracted from the WT and β2Ppo1 testes was treated with recombinant I-PpoI as indicated. Furthermore the PCR product (2kb) used as probe either treated with recombinant I-PpoI or untreated was analysed under the same hybridization conditions (lanes 4 and 5). Open and filled arrowheads indicate the full length and digested rDNA fragments respectively.

We injected mosquito embryos with the transformation construct pBac{3xP3-DsRed}β2-eGFP::I-PpoI that was designed to direct the expression of I-PpoI in the testis during the process of spermatozoa formation (Figure 1A). The structural properties of I-PpoI and eGFP allow the generation of a fusion protein that maintains both the activity and the selectivity of the endonuclease [18], while functioning as a visible marker for mosquito sexing during larval development. We utilized a shortened version of the β2 tubulin 5′ and 3′ regulatory regions [30] to direct expression of eGFP::I-PpoI. Previous studies have demonstrated that the β2 promoter is exclusively activated in male gonads and it can be utilised to selectively direct the expression of eGFP to the male gonads of anopheline mosquitoes [16]. The construct also contains piggyBac inverted repeats and the DsRed gene under the control of the 3xP3 promoter as a germline transformation marker (Figure 1A). Two independent transgenic lines (β2Ppo1 and β2Ppo2) were obtained in independent sets of embryo injections. Molecular analysis showed that each line resulted from a single integration event. Inverse PCR, followed by sequencing of the regions flanking the integration event, revealed that the construct had integrated at position 49029419 on chromosome 2L and position 11872203 on chromosome 3R in the lines β2Ppo1 and β2Ppo2, respectively (Figure S1). Both lines showed a strong green fluorescent signal localised in the male gonads, visible from late third instar larvae throughout adult development, thus indicating that the eGFP::I-PpoI fusion protein was exclusively expressed in the testes of male larvae, pupae and adults (Figure 1B).

To confirm this conclusion we searched for the presence of *in vivo* I-PpoI activity in the testes of β2Ppo mosquitoes. We analysed, by southern blot hybridization experiments, the integrity of the 28S rDNA genes in DNA extracted from β2Ppo1 mosquito testes (Figure 1C). Our results showed that, when using DNA from wild type mosquitoes, the probe containing the 28S rDNA sequence hybridized to a band of about 2.9 kb, in agreement with the size of the ribosomal gene and the position of the ClaI sites. Larger fragments were also recognized due to heterogeneity in the 28S ribosomal genes [18]. The same experiment carried out using DNA extracted from the testes of β2Ppo1 males showed the presence of a smaller band of 1.4kb, in agreement with the position of the I-PpoI recognition site in the ribosomal genes (Figure 1C and Figure S1). The same digestion product was observed when DNA extracted from wild type (WT) testes was treated with recombinant I-PpoI *in vitro* (Figure 1C). In addition, under *in vitro* conditions recombinant I-PpoI digested a 2kb PCR product encompassing the 28S rDNA probe fragment into a 1kb product. These results indicated that in the testes of transgenic males, the eGFP::I-PpoI fusion protein was able to cleave the 28S rDNA on chromosome X.

### β2Ppo Testes Develop Normally and Generate Spermatozoa Containing Active eGFP::I-PpoI

**Figure 2:**
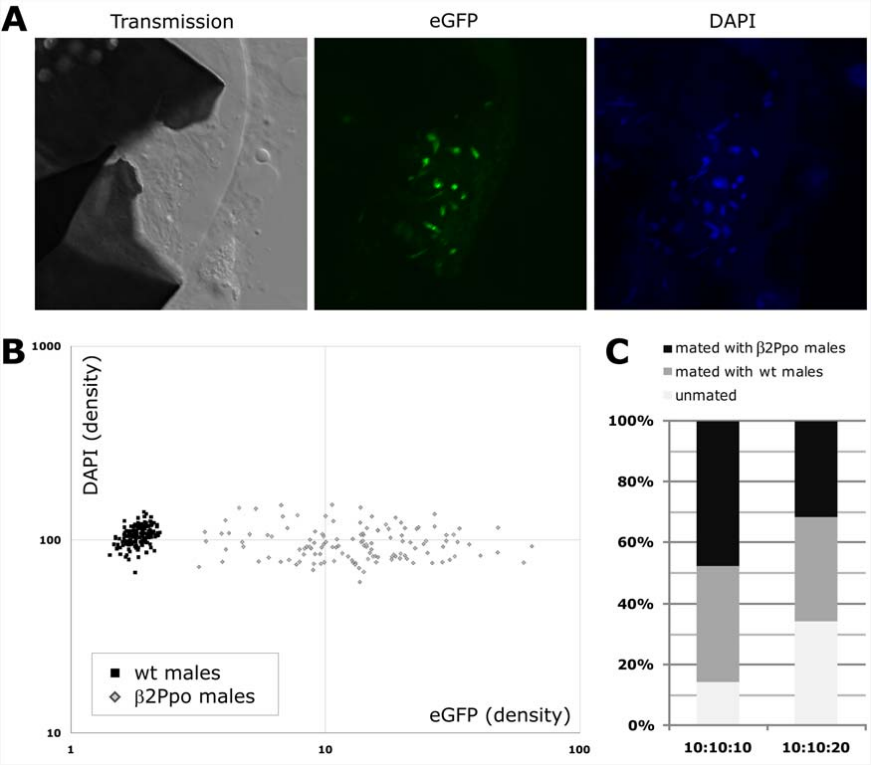
Confocal analysis of spermatozoa from β2Ppo and WT males recovered from spermathecae of WT females. (A) Spermatheca of a female mated with β2Ppo1 males analysed by transmission microscopy (left), analyzed for eGFP fluorescence (middle) and DNA stained with DAPI (right). (B) Analysis of confocal 3D data stacks of sperm extracted from spermathecae of females mated to WT or β2Ppo males. Objects defined as sperm, on the basis of DAPI fluorescence and size, were analyzed in a way that GFP density (nuclear volume/fluorescence intensity) was plotted against DAPI density. Density values were plotted for each individual spermatozoa examined from wt (black rectangles) and transgenic (grey diamonds) males. (C) Assessment of mating capability of β2Ppo2 against WT males. Equal numbers (10) of β2Ppo and WT males were allowed to mate in the presence of 10 or 20 WT females for 6 days. The mating with WT and transgenic males was assessed by analyzing in PCR experiments the DNA extracted from the spermathecae using a first a marker revealing chromosome Y specific sequence, to provide an overall estimate of mating rate and a second marker for the I-PpoI coding sequence. PCRs experiments that failed to amplify any product were scored as non-mated. The figure shows the percentage of mated mosquitoes and the relative contribution of WT (grey) and transgenic males (black) in the mating. Shown is the combined average of 3 independent sets of experiments.

We analyzed the development of the testes and spermatogenesis in heterozygous β2Ppo1 males using fluorescent microscopy and 3D imaging. Our results indicated that the testes were morphologically indistinguishable from those of wild type mosquitoes. The testes of β2Ppo1 mosquitoes showed a typical eGFP fluorescence pattern that reflected the direction of the differentiation process and the activational timing of the β2 tubulin promoter from sperm germ cells to mature spermatozoa. Confocal analysis of β2Ppo1 testes showed both mature sperm cells being produced in spermatocysts and spermatozoa reaching the male vas efferens (data not shown). In mating experiments with virgin wild type females, β2Ppo spermatozoa were found to be successfully transferred into the spermathecae (Figure 2A). All these spermatozoa showed a variable degree of green fluorescence localized to their nuclei, thus indicating they were carrying along substantial amounts of eGFP::I-PpoI fusion protein (Figure 2A). To investigate this phenotype in more detail we analyzed, by confocal microscopy and 3D-image reconstruction, the DNA content, the nuclear volume and the intensity of eGFP fluorescence of β2Ppo spermatozoa. Spermathecae from wild type females mated to either heterozygous β2Ppo2 or wild type males were dissected, fixed and stained with DAPI. Sperm were released and nuclei of about one hundred spermatozoa were individually analyzed for each cross. Wild type spermatozoa showed undetectable amounts of green fluorescence and a homogenous DAPI signal, whereas all spermatozoa from heterozygous β2Ppo2 males showed eGFP fluorescence signal which was mainly localized to the nuclei (Figure 2B). This analysis also indicated a moderate variability in the DAPI staining of β2Ppo spermatozoa (Figure 2B). The distribution of the eGFP fluorescence in β2Ppo spermatozoa is in agreement with both the transcription pattern of the β2 tubulin promoter in the testes and the structure of the spermatocyst: transcription from the β2 tubulin promoter starts shortly before the first meiotic division and continues throughout the subsequent stages of spermatozoa differentiation. Furthermore, in both insects and mammals, all spermatozoa derived from a single spermatogonial cell are connected through cytoplasmic bridges [31] to form a spermatocyst for a period of time that largely coincides with the temporal activity of the β2 tubulin promoter. This allows the sharing of cytoplasmic constituents between developing spermatozoa and would provide I-PpoI protein a means to migrate from cell to cell. Therefore, all spermatozoa, irrespective of whether they will inherit the transgene or not, are anticipated to carry along the eGFP::I-PpoI fusion protein.

### β2Ppo Heterozygous Males Induce Early Embryo Lethality

To investigate whether the expression of I-PpoI had an effect on fertility and/or sex ratio distortion of the progeny, we crossed heterozygote β2Ppo1 males with WT females. As a control, heterozygote β2Ppo1 females were crossed with WT males. Females were allowed to lay eggs on two consecutive occasions after they were blood fed. These experiments indicated that female β2Ppo1 mosquitoes did not show any anomalies in terms of fertility when crossed to WT males. These mosquitoes, compared to females of WT crosses, laid a normal number of eggs with comparable hatching rate, pupal development and adult sex ratio (Table 1 and data not shown). In contrast, while WT females crossed to β2Ppo1 males produced normal numbers of eggs, these eggs failed to hatch. These experiments were performed with β2Ppo1 mosquitoes of different generations (Table 1), as well as with line β2Ppo2 which showed identical properties (Table S1). Both transgenic lines, β2Ppo1 and β2Ppo2, have now been backcrossed to WT males for 14 and 16 generations respectively, and the males originating from these crosses were tested for fertility in each generation. Throughout this period no phenotype other than total male sterility was observed (data not shown).

**Table 1:** Outcome of crosses between transgenic β2Ppo and WT mosquitoes.

									Eggs laid	Larvae hatched	Screened	Transgenic	% Transgenic
**G2 crosses**	10	♂	**β2Ppo1**	**x**	30	♀	**wt**	**Lay 1**	658	0	-	-	-
								**Lay 2**	721	0	-	-	-
	20	♂	**wt**	**x**	17	♀	**β2Ppo1**	**Lay 1**	610	544	152	74	48.6%
								**Lay 2**	425	213	168	83	49.4%
**G3 crosses**	25	♂	**β2Ppo1**	**x**	25	♀	**wt**	**Lay 1**	971	0	-	-	-
								**Lay 2**	1669	0	-	-	-
	25	♂	**wt**	**x**	25	♀	**β2Ppo1**	**Lay 1**	1211	743	743	374	50.3%
								**Lay 2**	1331	1004	173	89	51.4%
	25	♂	**β2Ppo1**	**x**	25	♀	**wt**	**Lay 1**	713	0	-	-	-
								**Lay 2**	1694	0	-	-	-
	25	♂	**wt**	**x**	25	♀	**β2Ppo1**	**Lay 1**	1024	878	798	414	51.8%
								**Lay 2**	1424	1048	152	67	44.0%

Heterozygote β2Ppo1 males of generations 2 and 3 were crossed to WT females. As control β2Ppo1 heterozygote females of generation 2 and 3 were crossed to WT males. The total number of eggs laid and larvae hatched are shown for two consecutive egg depositions (Lay1 and Lay2). In addition larvae originating from control crosses were screened for the 3xP3-DsRed marker to determine the numbers of WT and transgenic offspring as indicated.

**Figure 3:**
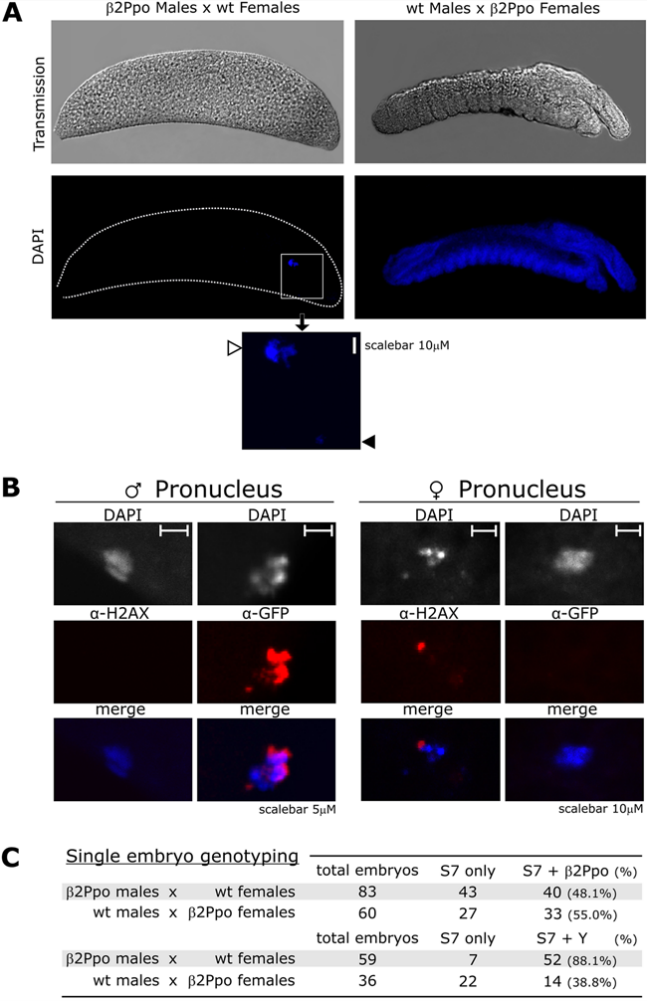
Morphological and genotype analysis of developmentally arrested embryos. (A) Embryos originating from crosses between β2Ppo males with WT females (left) compared to crosses between WT males and β2Ppo females (right) were analyzed by fluorescence microscopy 24 hours after oviposititon. The figure shows transmission (upper panels) and fluorescence of DAPI staining DNA (lower panels) of embryos oriented with the posterior end to the left and ventral side up. The inset shows a magnified view of the small and large DAPI stained bodies found in the embryos marked with a black and a white arrow respectively. (B) Immunostaining of freshly hatched embryos using mouse anti GFP (α-GFP) or mouse anti γ-H2AX (α-H2AX) primary in combination with anti-mouse IgG Alexa-532 conjugated secondary antibodies. DAPI stained bodies identified as male pronucleus and female pronucleus are shown at 5 and 10 µM scale respectively. (C) Molecular genotyping of embryos using multiplex PCR. Embryos originating from crosses of β2Ppo males with WT females and WT males with β2Ppo females were collected at 24 hrs post deposition and their DNA was examined using nested PCR analysis to amplify Y chromosome or transgene specific sequences as well as the ribosomal gene S7 as a control. The values show the frequency of the genotypes in all embryos that tested positive for the presence of S7.

To analyze the nature of male sterility in the β2Ppo1 and β2Ppo2 lines, we investigated whether the spermatozoa from these mosquitoes had fertilized the eggs in crosses with WT females and to establish the timing of embryo developmental arrest. For this purpose the embryos were fixed 24 hrs post oviposition, the chorion removed and the DNA stained with DAPI to highlight the localization and distribution of cell nuclei using confocal microscopy. In most of the embryos examined at 24 hrs after oviposition we could only identify two DAPI stained bodies. The first body was localized in the central anterior region of the embryo, while the second body was found in the anteroventral region in close proximity to the micropyle, thus suggesting their identification as the female and male pronuclei respectively (Figure 3A,B). A few embryos showed features of cellularization and nuclear division that did not progress to larvae formation. We also used confocal analysis to compare the size of sperm nuclei and the male pronuclei originating from β2Ppo males. This analysis showed that while the diameter of the sperm nuclei ranged from 2 to 4 µm, that of the male pronuclei was bigger (7–8 µm), suggesting that the latter had undergone decondensation. Male pronuclei were also stained with an antibody directed against eGFP (28 of 28 nuclei examined) while female pronuclei were found to stain with an anti γ-H2AX antibody (12 of 28 nuclei examined) that reacts with phoshorylated histone H2AX, associated with DNA double strand breaks [32]. This confirmed the transport of the fusion protein into the embryo and revealed the presence of DNA double strand breaks in the maternal genome (Figure 3B). Control experiments on WT embryos did not reveal nuclear reactivity with either the anti eGFP or the anti γ-H2AX antibody. On the basis of these findings, we concluded that β2Ppo males produced functional spermatozoa and that the observed sterility was the consequence of early embryo lethality mediated by I-PpoI activity on the chromosome X.

To study the genetic makeup of spermatozoa from heterozygous β2Ppo males we investigated the inheritance pattern of the transformation construct and the sex chromosomes in the developmentally arrested embryos. For this purpose we performed multiplex PCR experiments on single embryos using molecular markers revealing the presence of the Y chromosome, the transformation construct and, as a control, the ribosomal gene S7. When using primers specific for the I-PpoI open reading frame, we amplified a diagnostic band in 48% of embryos examined. This result showed that there was no bias in the inheritance of the transformation construct and indicated that non-transgenic spermatozoa carrying along the eGFP::I-PpoI fusion protein accounted for half of the embryo lethality induced by β2Ppo male mosquitoes. In other experiments we used a primerset designed to amplify a sequence that in previous reports was shown to specifically detect the *A. gambiae* Y chromosome [33]. Notably, in 88% of the embryos examined we amplified the diagnostic band for the Y chromosome, while control experiments carried out on embryos originating from β2Ppo females and WT males showed no such bias (Figure 3C). Although no viable progeny is produced in these crosses, this finding reveals a marked transmission ratio distortion towards the production of viable Y bearing spermatozoa in β2Ppo mosquitoes.

### Mating Competitiveness of β2Ppo Males in Laboratory Cage Experiments

With the aim of assessing the suitability of the β2Ppo1 and β2Ppo2 transgenic lines for SIT, we analyzed whether β2Ppo2 males could successfully compete with WT mosquitoes for mating partners in laboratory cage experiments. In these experiments identical numbers of WT and β2Ppo2 males were allowed to mate with varying numbers of WT virgin female mosquitoes. Five days later we collected the females and analyzed the spermathecae for the presence of either WT or β2Ppo spermatozoa in multiplex PCR experiments. We utilized a PCR primer pair amplifying the Y specific sequence to assess the mating rate of the females and a second primer pair amplifying the sequence of the I-PpoI transgene, to determine the number of females mated with β2Ppo2 males. Our data showed that the I-PpoI sequence could be amplified in a substantial proportion of female spermathecas, ranging from 48 to 56%, at different female to male ratios, thus suggesting that the transgenic males were not impaired in their ability to mate with WT females (Figure 2C).

## Discussion

We have generated two independent *A. gambiae* lines, β2Ppo1 and β2Ppo2, carrying the construct pBac{3xP3-DsRed}β2-eGFP-I-PpoI in distinct regions of the genome. Males originating from crosses between heterozygous β2Ppo females and WT males showed, starting from late 3^rd^ instar larvae, a strong green fluorescence signal exclusively localized in the testes, indicating that the eGFP::I-PpoI fusion protein was being produced in spermatozoa according to the anticipated expression pattern of the β2 tubulin promoter. Our results also demonstrated that in the testes of β2Ppo males ribosomal DNA was cleaved at the I-PpoI site. This finding indicated that the endonuclease component of the eGFP::I-PpoI fusion protein retained its ability to cut the X chromosome *in vivo.* Intriguingly, heterozygous β2Ppo males were completely sterile. To understand the molecular basis of this phenotype, we investigated whether the expression of I-PpoI disrupted the process of spermatogenesis or impaired the ability of spermatozoa to enter eggs. Microscopy analysis indicated that the testes of β2Ppo males produced spermatozoa morphologically identical to those of WT mosquitoes. Dissection of female mosquitoes mated with β2Ppo males indicated that the spermatozoa had been successfully transferred to the spermathecae. Furthermore, confocal microscopy showed the presence of both the female and male pronuclei in the embryos. Taken together, these experiments demonstrate that β2Ppo males produced competent and viable spermatozoa. Nuclear fluorescence staining also revealed that the embryos originating from β2Ppo males were arrested very early in their development, probably at a point before the fusion of the male and the female pronuclei.

The genetic study of these embryos provided clues to formulate a molecular explanation for the early dominant lethality induced by β2Ppo males. Although spermatozoa carrying the transgene had fertilized only half of the eggs, as inferred by PCR genotyping analysis, confocal microscopy and 3D imaging demonstrated that all spermatozoa showed some level of eGFP fluorescence. This observation is in agreement with the temporal expression of the β2tubulin promoter and the structure of the spermatogonial syncytium. Importantly, it also indicates that all spermatozoa of β2Ppo males could deliver active nuclear I-PpoI protein into the embryos, thereby inducing DNA damage to the maternal inherited chromosome X. This provides an explanation for the dominance of the lethality phenotype. This notion was also supported by findings showing that individual male nuclei within the developmentally arrested embryos stained positive with anti-eGFP antibodies, while the female pronuclei did not. In contrast antibodies directed against γ-H2AX indicated double strand DNA damage only on the female pronuclei.

The genetic analysis of the embryos also revealed that more than 80% had originated from spermatozoa carrying the Y chromosome, thus indicating that although the expression of eGFP::I-PpoI did not impair the process of spermatogenesis or the viability of sperm cells in general, it did selectively target X chromosome carrying spermatozoa, thereby causing transmission ratio distortion. It is possible that the remaining embryos had been fertilised by spermatozoa lacking both chromosomes X and Y. Although of no immediate practical application due to embryo lethality, these results demonstrate that synthetic sex distortion mechanisms can be developed. Both mathematical modelling and the study of naturally occurring sex distorters in some insect species predict that, if linked to the Y chromosome, such distorters would represent extremely powerful tools to knock down a target population in a relatively short time.

The development of the transgenic lines β2Ppo1 and β2Ppo2 has some direct implications for the implementation of vector control measures based on genetically modified mosquitoes. Both lines meet a number of desirable requirements for SIT, including: i) a visible marker for monitoring male dispersal and competitiveness, ii) a validated sexing system that can be effectively automated; and iii) complete and dominant genetic male sterility. Laboratory cage experiments performed here indicate that β2Ppo male mosquitoes are not impaired in their ability to mate with WT females when mixed with WT males. Since the I-PpoI recognition site is located in the 28S rDNA gene in a highly conserved rRNA region, which forms the peptidyl transferase centre of the ribosome, the approach described here could be applied to other pest species.

The finding that non-genetically modified spermatozoa can carry along effector molecules selectively targeting the maternal genome offers the possibility to develop “Medea”- like cytoplasmic incompatibility systems predicted to have strong driving properties. Finally, our results reveal the intriguing possibility of manipulating maternally inherited mosquito genes involved in parasite transmission or sex determination by using wild type sperm cells carrying engineered endonucleases such as HEGs or zinc fingers. To this end, heterozygous transgenic males can be produced that express, during the process of spermatogenesis, a rare-cutting endonuclease engineered to selectively target such genes. Our findings demonstrate that endonuclease protein will be transferred to all spermatozoa irrespectively of the segregation of the transgene itself and therefore will be transported into the wild type embryo at the time of fertilization. The endonuclease will introduce changes into the targeted maternal sequence by cleavage followed by non-homologous repair. A fraction of the embryos will inherit these endonuclease-induced changes without carrying the original transformation construct. The resulting mosquitoes would address a number of safety and environmental issues associated with the release of genetically manipulated mosquitoes for vector control as they will not contain a selectable marker or a transformation construct.

## Materials and Methods

### Plasmid Construction

The 1.2kb eGFP::I-PpoI cassette was amplified from pEGFP-nPpo [18] using primers PpoH34b2f, ACCGGTCAAGCTTATGGTGAGCAAGGGCGAGGAGCTGTTC and PpoH34b2r GGTACCGTCAAGCTTATACCACAAAGTGACTGCCCCTTTGTTG. A 1.7kb beta2 tubulin GFP cassette was amplified from pPB[DsRed]beta2EGFP [16] using primers b2sAscIfwd AAGGCGCGCCCTAGCGTTCATAATTGATATAG and b2sAscIrev AAGGCGCGCCCGATTTAAGGACCGATTCC and cloned into the shuttle vector pSLfa1180fa [34] using AscI. From this vector the original GFP was removed with HindIII and replaced by the eGFP::I-PpoI cassette cut with HindIII. The resulting 2.3kb cassette contains the nuclear localization signal between the N-terminal eGFP and the C-terminal I-PpoI coding regions which are flanked by the β2 regulatory regions and was moved into the pPB[DsRed] backbone using AscI to create pBac{3xP3-DsRed}β2-eGFP::I-PpoI.

### Development of Transgenic Lines

Transgenic lines were developed as described [16],[35]. *A. gambiae* (strain G3) embryos were injected using a Femtojet Express injector and sterile Femtotips (Eppendorf) with a mixture of 0.2 µg/µl of plasmid and 0.8 µg/µl of piggyback helper RNA. The hatched larval survivors were screened for transient expression of the 3xP3 DsRed marker and only transients were grown up and crossed to wild-type mosquitoes. The progeny of these crosses was analyzed for DsRed fluorescence. To establish line β2Ppo1 we injected 430 embryos from which 42 (9.7%) survivors hatched 21 (50%) of which showed transient expression of the marker. 6 female transients survived to adulthood and when crossed to WT gave rise to 1 transgenic female individual. To establish β2Ppo2 we injected 241 embryos and obtained 45 (18.6%) survivors including 30 transients (66.6%). 11 female transients survived to adulthood and when crossed to WT gave rise to 8 transgenic individuals (2 males, 6 females) from one founder. Females were crossed separately to WT males and molecular analysis of their progeny confirmed that they had originated from a single integration event. Transgenic mosquitoes at different developmental stages were analyzed on a Nikon inverted microscope (Eclipse TE200) to detect eGFP and DsRed expression. Digital images were captured on a Nikon inverted microscope (Eclipse TE200) with an attached Nikon DXM1200 digital camera. The β2Ppo lines were reared in a way so that in each generation transgenic mosquitoes were separated into males and females and crossed back to WT *A. gambiae* G3.

### Southern Blot

Genomic DNA was digested with ClaI in the presence and absence of I-PpoI. As a probe we used a 2 kb rDNA PCR fragment amplified from genomic DNA using the primers rDfwd GCCGAAGCAATTAGCCCTTAAAATGGATG and rDrev CACCAGTAGGGTAAAACTAACCTGTCTCACG. The probe was labelled with P^32^ using the High Prime DNA labelling kit (Roche) and purified with ProbeQuantTM G-50 columns (GE Healthcare). Results were visualized using a FUJIFILMFLA-5000 Phosphoimager (Fuji Photo Film Co. Ltd, Stamford, CT, USA). For in vitro digestions, we used commercially available I-PpoI (Promega) enzyme.

### Analysis of Sperm Nuclei Recovered from Spermatheca

Virgin females mated with WT males or transgenic males were dissected in PBS. Spermathecae were checked for the presence of sperm on a widefield microscope. Spermathecae containing sperm were fixed in methanol–free 4% formaldehyde (Pierce) in PBS for 30min, washed 3 times for 15min in 0.1% Tween-20 PBS and transferred on a fresh slide containing Vectashield mounting medium with DAPI (Vectorlabs. Inc.). Cover slips were added to gently crack the spermathecae and release sperm. Samples in which the sperm nuclei were sufficiently diluted were then subjected to further analysis. Multiplane z-series were collected with a confocal microscope (SP5; Leica) and a 23× lens. Confocal microscope z-series were analyzed using image-analysis software (Volocity; Improvision Inc.). Stacked images were used to render 3D reconstructions of the sperm nuclei. Objects were defined on the basis of DAPI fluorescence intensity and by size, and were then measured for DAPI and GFP density (intensity/volume).

### Embryo Fixation and Nuclear Staining

Sterile embryos were collected from crosses of β2Ppo males mated with WT virgin females and control embryos from crosses of WT males with β2Ppo females. Females were allowed to egg-lay 48 hrs post blood-feeding. The exochorion of up to 24 hrs old embryos was removed and embryos fixed essentially as described [36]. Fixed embryos were stored at −20°C in methanol. To stain nuclei, the endochorion was gently peeled off by submerging embryos on double side tape in methanol and gently stroking them out using a fine brush. Embryos were rehydrated in PBTA (1× PBS, 1% BSA, 0.05% Triton X-100, 0.02% Sodium Azide) for 15min on a rotator. DNA was stained for 15min in the dark with DAPI (1 µg/ml) and washed twice for 1 hour and once overnight with fresh PBTA avoiding unnecessary light exposure. Embryos were then mounted on slides and subjected to confocal analysis (SP5; Leica).

### Immunohistochemistry

Rehydrated embryos were probed with mouse monoclonal anti γ-H2AX (Ser139 mouse monoclonal; Upstate Biotechnology; 1∶200) to detect the phosophorylated form of histone H2AX. Alternatively embryos were probed with mouse monoclonal anti-GFP (Living Colours JL-8; 1∶200). Embryos were probed overnight at 4°C and then washed 3 times and once for one hour in PBTA. As secondary antibody we used goat anti-mouse IgG Alexa-532 conjugate (Molecular Probes; 1∶500) and washed as described above. Embryos were then mounted on slides for confocal analysis in Vectashield containing DAPI (Vectorlabs. Inc).

### Single Embryo Genotyping

Embryos were homogenized in 5 µl extraction buffer (10 mM Tris, pH 8.2; 1 mM EDTA; 25 mM NaCl) containing 200 µg/ml proteinase K (Sigma) and incubated for 1h at 37°C followed by 10min at 95°C. The whole extraction was then used in a 25 µl outer PCR reaction using the Phusion Hotstart DNA polymerase (Finnzymes). 0.5 µl of this PCR was used in an inner reaction with the same conditions: (35sec at 98°C; 35 rounds of 15sec at 98°C, 40sec at 61°C, 30sec at 72°C; and 5 min at 72°C). The nested primes used were: S7IF, GGCGATCATCATCTACGTGC; S7OF, GAATCGAACTCTGGTGGCTGA and S7OR, CTTTTCTGCGTCCACCCCGA; S7IR, GTAGCTGCTGCAAACTTCGG for the amplification of the S7 control gene. Primers mag-mdg1IF, ATGTAGCATGTGGAGCAGTTC; mag-mdg1OF, CATACTAACAACTGATGCTTCAGATG and mag-mdg1IR, GCTCTTTGAGGATGGCAAC; mag-mdg1OR, CGCGTTGTTTTCGGTTTGCA were used to check for the presence of the Y chromosome [33]. Primers PPoIF, CGACCTAAGAAGAAGAGGAAGGTGA; PPoOF, GAGCTGTACAAGTCCGGACTCAGA; and PpoIR, CTTTGTTGAGGACCTGCCACAGT; PpoOR, CTTATACCACAAAGTGACTGCCCCT amplify the I-PpoI open reading frame to check for presence of the transgene.

## Supporting Information

Figure S1 Location of transgene integration sites and genomic rDNA repeats. Positions of insertions are shown as well as the 14 basepairs flanking the transformation constructs on each side (lower right panel). The structure of the rDNA repeat unit including the 3 ribosomal genes and the internal transcribed spacers (ITS) as well as a detailed view of the 28S rDNA gene around the I-PpoI recognition site is shown in the upper right panel. Primers rDfwd and rDrev were used to generate the 2kb probe for southern hybridization.
(0.42 MB TIF)

Table S1 Outcome of crosses between transgenic β2Ppo and WT mosquitoes. Heterozygote β2Ppo2 males of generation 3 were crossed to WT females. As control β2Ppo2 heterozygote females of generation 3 were crossed to WT males. The total number of eggs laid and larvae hatched are shown for two consecutive egg depositions (Lay1 and Lay2). In addition larvae originating from control crosses were screened for the 3xP3-DsRed marker to determine the numbers of WT and transgenic offspring as indicated.
(0.05 MB JPG)
