# Supplementary figures and images for: Targeting the X Chromosome during Spermatogenesis Induces Y Chromosome Transmission Ratio Distortion and Early Dominant Embryo Lethality in Anopheles gambiae
Source: PLoS Genet. 2008 Dec 5;4(12):e1000291. doi: 10.1371/journal.pgen.1000291 (PMC2585807; doi:10.1371/journal.pgen.1000291)

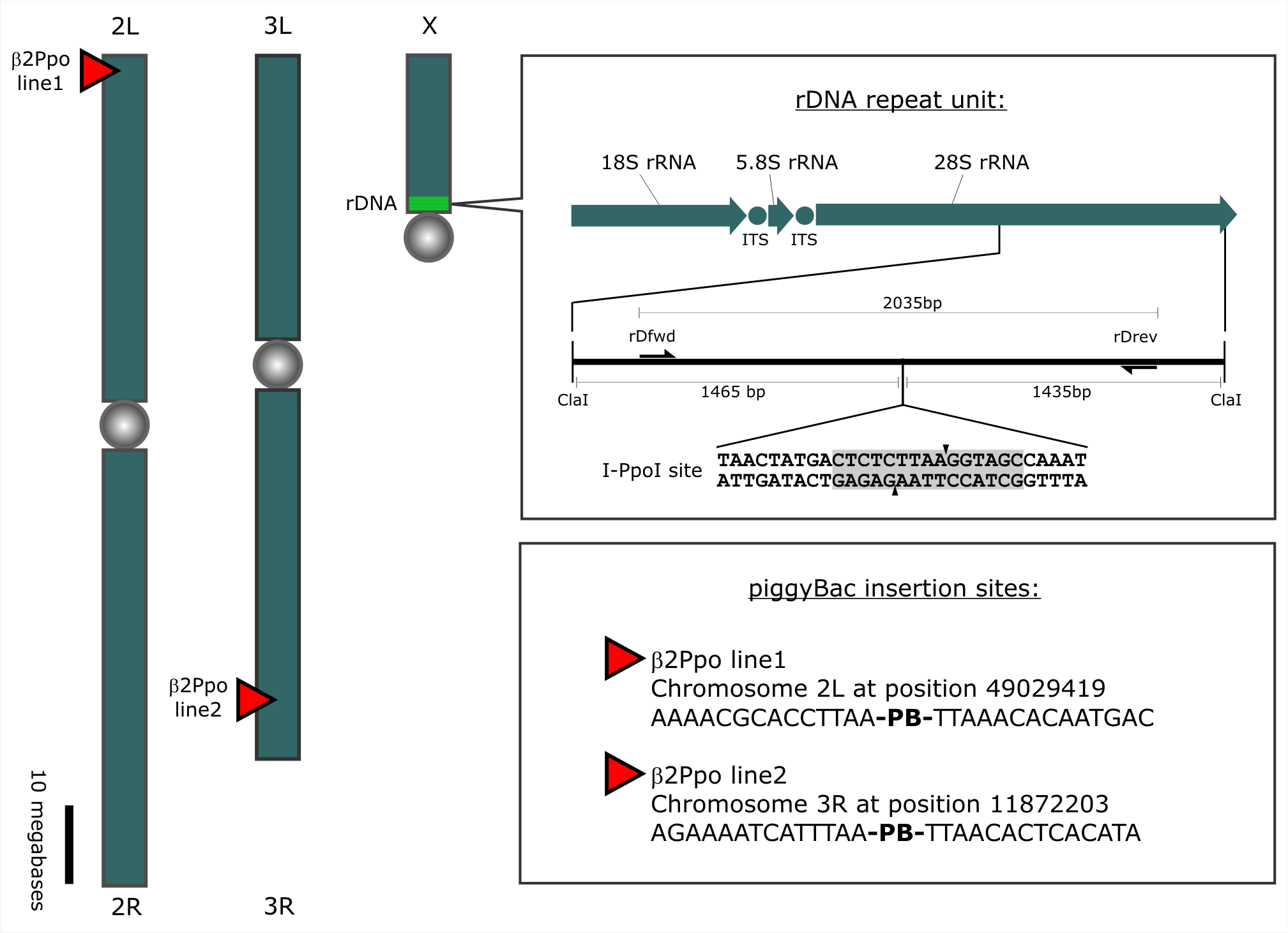

Supplement: Figure S1 — Location of transgene integration sites and genomic rDNA repeats. Positions of insertions are shown as well as the 14 basepairs flanking the transformation constructs on each side (lower right panel). The structure of the rDNA repeat unit including the 3 ribosomal genes and the internal transcribed spacers (ITS) as well as a detailed view of the 28S rDNA gene around the I-PpoI recognition site is shown in the upper right panel. Primers rDfwd and rDrev were used to generate the 2kb probe for southern hybridization. (0.42 MB TIF) [file pgen.1000291.s001.tif]

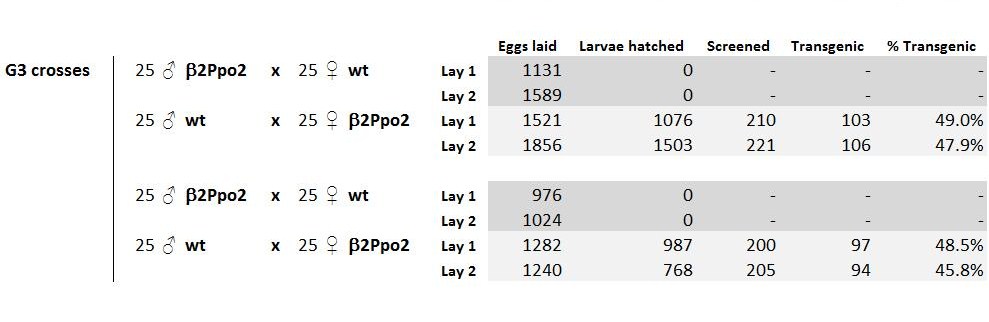

Supplement: Table S1 — Outcome of crosses between transgenic β2Ppo and WT mosquitoes. Heterozygote β2Ppo2 males of generation 3 were crossed to WT females. As control β2Ppo2 heterozygote females of generation 3 were crossed to WT males. The total number of eggs laid and larvae hatched are shown for two consecutive egg depositions (Lay1 and Lay2). In addition larvae originating from control crosses were screened for the 3xP3-DsRed marker to determine the numbers of WT and transgenic offspring as indicated. (0.05 MB JPG) [file pgen.1000291.s002.jpg]
